# Detection limit of intragenic deletions with targeted array comparative genomic hybridization

**DOI:** 10.1186/1471-2156-14-116

**Published:** 2013-12-05

**Authors:** S Hussain Askree, Ephrem LH Chin, Lora H Bean, Bradford Coffee, Alice Tanner, Madhuri Hegde

**Affiliations:** 1 Medical Neurogenetics LLC, 5424 Glenridge Drive, Atlanta, GA, USA; 2 Oxford Gene Technology, Baylor College Medicine, 520 white Plains Road, Tarrytown, NY 10591, Houston, TX, USA; 3 Emory Genetics Laboratory, Department of Human Genetics, Emory University, 2165 N Decatur Road, Decatur, GA 30033, USA

**Keywords:** aCGH, Intragenic deletions, Breakpoint analysis, Molecular characterization

## Abstract

**Background:**

Pathogenic mutations range from single nucleotide changes to deletions or duplications that encompass a single exon to several genes. The use of gene-centric high-density array comparative genomic hybridization (aCGH) has revolutionized the detection of intragenic copy number variations. We implemented an exon-centric design of high-resolution aCGH to detect single- and multi-exon deletions and duplications in a large set of genes using the OGT 60 K and 180 K arrays. Here we describe the molecular characterization and breakpoint mapping of deletions at the smaller end of the detectable range in several genes using aCGH.

**Results:**

The method initially implemented to detect single to multiple exon deletions, was able to detect deletions much smaller than anticipated. The selected deletions we describe vary in size, ranging from over 2 kb to as small as 12 base pairs. The smallest of these deletions are only detectable after careful manual review during data analysis. Suspected deletions smaller than the detection size for which the method was optimized, were rigorously followed up and confirmed with PCR-based investigations to uncover the true detection size limit of intragenic deletions with this technology. False-positive deletion calls often demonstrated single nucleotide changes or an insertion causing lower hybridization of probes demonstrating the sensitivity of aCGH.

**Conclusions:**

With optimizing aCGH design and careful review process, aCGH can uncover intragenic deletions as small as dozen bases. These data provide insight that will help optimize probe coverage in array design and illustrate the true assay sensitivity. Mapping of the breakpoints confirms smaller deletions and contributes to the understanding of the mechanism behind these events. Our knowledge of the mutation spectra of several genes can be expected to change as previously unrecognized intragenic deletions are uncovered.

## Background

Laboratories that offer diagnostic mutation testing use a number of methodologies to detect pathogenic chromosomal rearrangements, coding sequence aberrations, abnormal methylation patterns, and other biochemical indicators of genetic disease. These analyses help with diagnoses, management, carrier testing, and counseling for families affected by an inherited genetic disease. The mutation spectrum of a particular gene guides clinical test development, so that the adapted method promises the highest yield in detection without compromising sensitivity, specificity, and cost effectiveness. Small mutations, such as nucleotide changes predicted to cause missense, nonsense, or altered splicing, as well as frameshifts due to small deletions and duplications of a few bases, can be detected by sequence analysis. Larger pathogenic copy number variations (CNVs) are efficiently detected by high-resolution G-banding, fluorescence *in situ* hybridization (FISH), and cytogenomic array comparative genomic hybridization (aCGH); however, the size limitation of these methods is approximately 200–500 kb or larger.

Recurrent microdeletions and microduplications that occur between repeat sequences via nonallelic homologous recombination (NAHR) are a class of large pathogenic CNVs that can easily be detected in diagnostic tests, as the known breakpoints are amenable to the development of targeted methods [1,2]. On the other hand, there are CNVs that primarily represent private non-recurrent familial mutations encompassing several to a single gene. Nonhomologous end-joining (NHEJ) and microhomology-mediated break-induced replication (MMBIR) are two mechanisms responsible for these mutations [3-5]. Chromosomal microarray is the recommended technique to screen the entire genome for CNVs, when there is no specific locus clinically suspected [6].

Gene-targeted diagnostic testing methods can be developed to screen a specific genomic locus for CNVs, which is best illustrated by the diagnostic testing for Duchenne muscular dystrophy [7]. Pathogenic deletions and duplications within the *DMD* gene account for approximately 65 percent of mutations. Clinical testing for these mutations has been performed by multiplex standard PCR (males only) [8,9], quantitative PCR (q-PCR) [10], and Southern blotting [11], as well as multiple ligation-dependent probe amplification (MLPA) [12]. These methodologies are laborious and lack sensitivity, particularly for females, often requiring confirmation testing by a second method. To date, the most cost-effective and sensitive method for the detection of mutations in Duchenne muscular dystrophy is array-based comparative genomic hybridization (aCGH) [7,13,14].

Several gene-targeted arrays have been developed with probes concentrated within loci of interest. Examples include an aCGH that targets regions with segmental duplications and arrays that target only 5–8 specific genes of interest [15-18]. To be useful in a diagnostic laboratory, the design of aCGH has to be optimized to yield coverage of as many disease-associated genes as possible without compromising resolution and sensitivity to detect small intragenic pathogenic CNVs. Roughly, the detection criteria can be considered single or multiple exonic CNVs. Detection of pathogenic CNVs at sub-kilobase resolution have been reported by our laboratory and Boone et al. (2010), illustrating the ability of this technology to identify mutations in patients with various diseases and syndromes [19,20]. We have previously reported the development of a custom exon-centric array designed and implemented at Emory Genetics Laboratory (EGL) [20]. We now report aCGH data from a set of representative deletions identified with the use of these arrays during routine laboratory testing that demonstrate the power and sensitivity of this technology and illustrate the limit of detection in terms of deletion size (Table 1).

**Table 1:** Table lists all the cases with intragenic deletions discussed in this manuscript

**Case**	**Gene**	**Disease: Inheritance**	**Mutation detected with sequencing**	**Mutation detected with aCGH**	**Deletion size**
**1**	*BCKDHB*	Maple syrup urine disease: AR	c.596_597delGT	Exon 9 deletion	~58 kb
**2**	*FH*	Hereditary leiomyomatosis and renal cell cancer: AD		Exons 2–9 deletion	~19 kb
**3**	*DBT*	Maple syrup urine disease: AR	c.871C > T (p.R291X)	Exon 5 deletion	~3.7 kb
**4**	*HPRT1*	Lesch-Nyhan syndrome: XL		Exon 5 deletion	2,319 bp
**5**	*STK11*	Peutz-Jeghers syndrome: AD		Exon 8 deletion	1,325 bp
**6**	*STK11*	Peutz-Jeghers syndrome: AD		Exon 3 deletion	971 bp
**7**	*PAH*	Phenylketonuria: AR	c.838G > A (p.E280K)	Partial exon 6 deletion	801 bp
**8**	*EMD*	Emery-Dreifuss muscular dystrophy: XL		Exon 2 deletion	267 bp
**9**	*DBT*	Maple syrup urine disease: AR		Partial exon 11 deletion	> 3.5 kb
				c.344-4del12	12 bp
**10**	*POMT1*	Walker-Warburg syndrome AR	c.2167dupG	No deletion: c.160_161ins349	False positive: Alu insertion
**11**	*SLC9A6*	X-linked intellectual disability: XL		No deletion	False positive: hemizygous missense
**12**	*GALT*	Galactosemia: AR	c.855G > T (p.K285N) mutation & c.844C > G (p.L282V) variant	No deletion	False positive: compound heterozygous missense

AD: Autosomal Dominant; AR: Autosomal Recessive; XL: X-linked.

## Results

### Univocal detection of deletions larger than 2 kb

**Figure 1:**
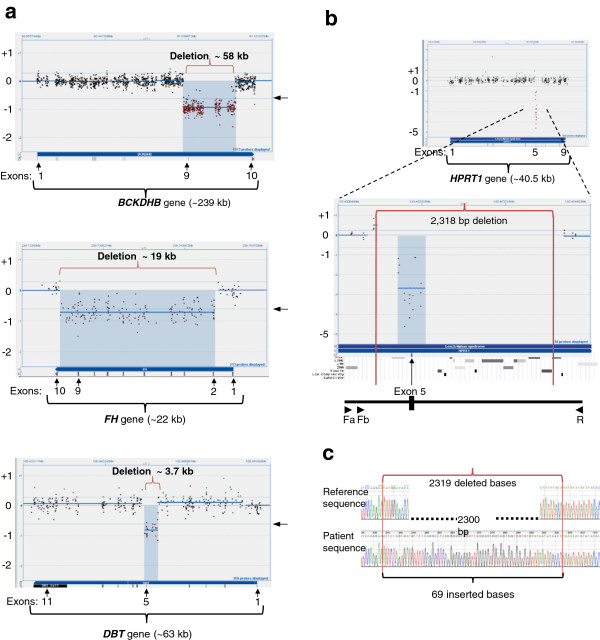
**Examples of large deletions (>2 kb).** CytoSure display of aCGH data across genes, with exon locations marked below. The patient versus reference Cy3/Cy5 ratio is plotted for each sample. Shown are the mean of the log2 ratio (thick blue horizontal line) and thresholds for deletion and duplication calls at log2 ratios of +0.4 and -0.6, respectively (thin blue horizontal lines), derived from the CBS algorithm. Arrows on the right of the CytoSure display mark the -0.6 log2 threshold for deletions. Below the CytoSure display are the corresponding exon tracks. **1a** top) ~58-kb deletion encompassing exon 9 of the *BCKDHB* gene. **1a** middle) ~19-kb deletion encompassing exons 2–9 of the *FH* gene. 1a bottom) ~3.7-kb deletion encompassing exon 5 of the *DBT* gene. **1b**) 2,318-bp deletion encompassing exon 5 of the *HPRT1* gene. The breakpoints of the deletion are shown with vertical red lines. Below the CytoSure display are the UCSC RepeatMasker track showing repeat elements at the deletion locus, followed by an illustration of the breakpoint PCR design with location of primers (Fa, Fb, and R) shown as arrows. **1c**) Electropherogram of sequenced *HPRT1* deleted allele with 69 inserted bases shown. Image in Figure 1c was reproduced, with kind permission of Springer Science + Business Media, as previously published in Molecular Genetics and Personalized Medicine, edited by Best DH and Swensen JJ, Jan 2012, Chapter 2: Array Comparative Genomic Hybridization in Cytogenetics and Molecular Genetics, Askree SH and Hegde M, Figure 2.5, Page 32.

Custom-designed gene-targeted aCGH, manufactured on an Agilent aCGH platform developed by OGT’s Genefficiency service (Oxford Gene Technology, Oxford, UK), was used for deletion and duplication mutational analysis for genes that are part of our laboratory’s sequence analysis repertoire [20]. The Circular Binary Segmentation (CBS) algorithm generated deletion calls using a log2 ratio of each segment that has a minimum of four probes [21]. Threshold factor for deletions was set as a log2 ratio of -0.6. Figure 1 shows several examples of aCGH data with intragenic deletions larger than 2 kb that were easily detected with CBS calls that crossed the -0.6 log2 ratio threshold. Sequence analysis of the three maple syrup urine disease (MSUD) genes, *BCKDHA*, *BCKDHB*, or *DBT*, in two unrelated cases of known biochemical diagnosis of MSUD detected only one familial mutation each (Table 1) [22]. One had a known pathogenic mutation in the *BCKDHB* gene (c.596_597delGT), whereas the other family had a *DBT* gene nonsense mutation (c.871C > T (p.R291X). Reflex testing for with aCGH yielded the corresponding second familial mutation; an approximately 58-kb deletion encompassing exon 9 of the *BCKDHB* gene (Figure 1a top) and an approximately 3.7-kb deletion encompassing exon 5 of the *DBT* gene were detected in the two above mentioned MSUD cases (Figure 1a bottom). These examples demonstrate how conclusive a single exon deletion can be when several probes target the deleted sequence within the breakpoints and the CBS call is well below the log2 ratio threshold. Parental samples were tested to confirm biallelic inheritance in the probands of both families. Finding two familial mutations in this autosomal recessive disorder made carrier-testing possible for other at-risk family members.

An additional example presented here is where aCGH analysis of the *FH* gene detected a heterozygous deletion of an intermediate size compared to the two single exon deletions detected in MSUD cases described above. However, this 19-kb deletion resulted in a loss of 8 out of 10 exons of the *FH* gene. This testing was triggered due to strong clinical suspicion, in an adult male with a personal and family history that was highly suggestive of the autosomal dominant disorder, hereditary leiomyomatosis and renal cell cancer. Sequencing of the relevant *FH* gene did not detect a mutation, and aCGH analysis confirmed the familial (autosomal dominant) *FH* deletion mutation (Figure 1a middle).

In contrast to the deletions detected in autosomal genes, deletions in X-linked diseases show high sensitivity in male probands due to lack of an interfering normal allele. Lesch-Nyhan syndrome (LNS) is an X-linked recessive disorder caused by deficiency of the enzyme hypoxanthine guanine phosphoribosyltransferase (HPRT). A mutation in the single copy of the *HPRT1* gene in a male causes LNS.[23] A 20-year-old male proband was found to carry a 2.3-kb hemizygous deletion mutation encompassing exon 5 in the *HPRT1* gene (Figure 1b). Subsequently, his sister was found to carry the familial mutation. We tested amniocytes from the sister’s pregnancy and determined that the fetus did not inherit the familial deletion mutation. Allele-specific PCR was developed that amplified the deleted allele in the proband and his sister. Sequence analysis confirmed a 2319-bp deletion encompassing exon 5 with breakpoints at the exon 5 splice site boundary (Figure 1b, 1c). There was an insertion of 69 bp with no homology to any flanking sequence. Upon BLAT query, the inserted bases mapped to chr5p13.1 (Chr5:40,844,202-40,844,270/hg18) [24]. Data included in additional information shows the aCGH analyses on all three family members, the fragment analysis of the breakpoint PCR, as well as the complete sequence of the deletion locus (see Additional file 1).

### 1325-bp and 971-bp deletions in the STK11 gene

**Figure 2:**
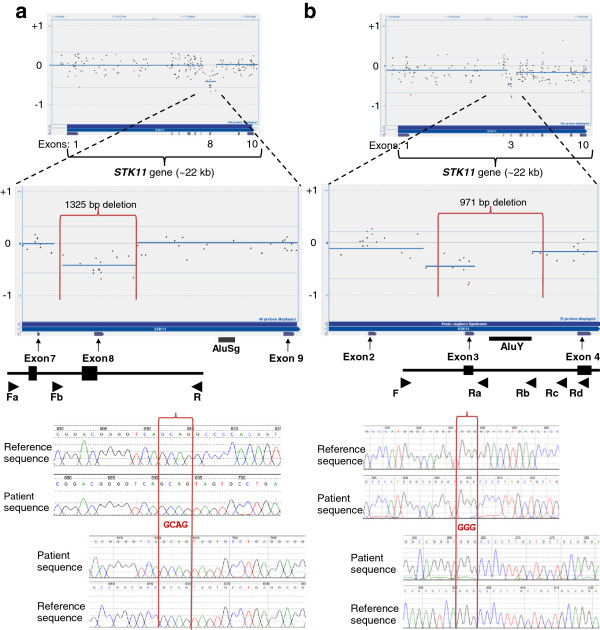
**Single exon deletions in the *STK11 *gene.** Figure 2 shows the data from two independent aCGH analyses with probes targeting the entire *STK11* gene, and the zoomed-in view of where the deletion was present. CBS-generated deletion call did not cross the -0.6 threshold in either analysis. The breakpoints of the actual deletion are shown with vertical red lines. Below the CytoSure display are the corresponding exon tracks. Locations of Alu elements in the region of the deletion are marked. At the bottom is an illustration of the breakpoint PCR design, with the location of respective primers shown as arrows. Electropherograms of bidirectionally sequenced deleted alleles are shown with sequencing in the forward direction on top and reverse sequencing below. The four- and three-base pair microhomology at the breakpoints are shown within the two vertical red lines that demarcate the breakpoints. **2a**) 1325-bp deletion encompassing exon 8 of the *STK11* gene, with electropherogram of sequenced *STK11* across deleted exon 8. **2b**) 971-bp deletion encompassing exon 3 of the *STK11* gene, with electropherogram of sequenced *STK11* across deleted exon 3.

Peutz-Jeghers syndrome (PJS) is an autosomal dominant disorder characterized by intestinal hamartomatous polyps, an increased risk of certain malignancies, and hyperpigmented cutaneous lesions. Mutations in the *STK11* gene cause PJS. In two unrelated Caucasian patients who had clinical presentations consistent with PJS, sequence analysis of the *STK11* gene did not detect a mutation.[25] Figure 2 shows aCGH data where a deletion call encompassing exon 8 did not cross the -0.6 log_2_ ratio threshold set. However, low hybridization of 15 probes leading to a deletion call of a >1-kb segment triggered PCR confirmation. Using primers flanking the breakpoint, the deleted allele was preferentially amplified over the larger normal allele. Sequencing data confirmed a 1325-bp deletion with a four-base microhomology at the breakpoints in intron 7 and intron 8 (Figure 2a). Fragment analysis of breakpoint PCR, and the complete sequence of the deletion locus is included in Additional file 2.

In the second PJS patient, a deletion call encompassing exon 3 did not cross the -0.6 log_2_ ratio threshold set, but was appreciated in manual review (Figure 2b). In contrast to the previous case, the call was based on 9 probes. However, the patient’s clinical presentation, as reported by the referring physician, was highly suggestive of PJS syndrome. A 971-bp deletion encompassing exon 3 of the *STK11* gene was subsequently confirmed and breakpoints mapped with allele-specific PCR and sequencing (see additional file 2). Several probes that map within the deletion did not show hybridization ratios, as would be expected with the deletion in one allele.

### 801-bp deletion resulting in partial deletion in the PAH gene

**Figure 3:**
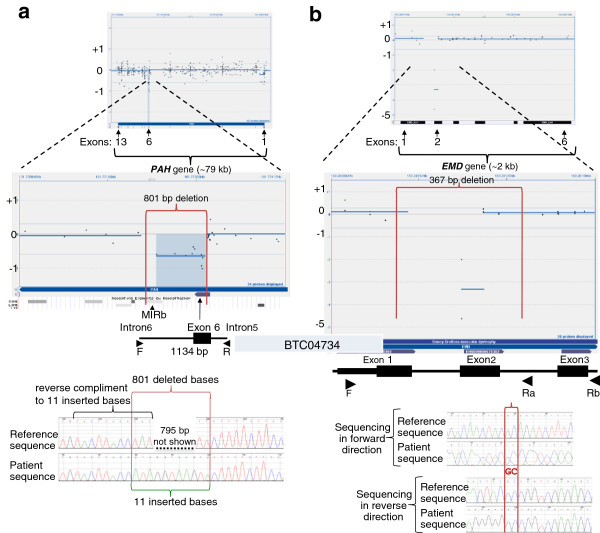
**Deletions with a breakpoint in exons.** Figure 3 shows CytoSure display of aCGH data with probes targeting the *PAH* gene and *EMD*. The top panel displays the entire gene view, and the lower panel zooms into the region showing a deletion. Below the CytoSure display are the corresponding exon tracks, and the UCSC RepeatMasker track showing repeat elements at the deletion locus. Vertical red lines mark the breakpoints. At the bottom is an illustration of the PCR design. Primers are shown as arrows. **3a**) 801-bp deletion encompassing part of exon 6 of the *PAH* gene, with electropherogram of sequenced *PAH* gene across deletion. The breakpoints are shown with vertical red lines. The inserted 11 bases, shown with a green bracket, correspond to the reverse compliment of the bases labeled with a black bracket in the reference sequence across one of the breakpoints. **3b**) 367-bp deletion encompassing part of exon 1 and all of exon 2 of the *EMD* gene, with electropherogram of sequenced *EMD* gene across deletion. The two-base pair microhomology at the breakpoints is shown within the two vertical red lines that demarcate the breakpoints.

A sample from a biochemically diagnosed 6-year-old phenylketonuria (PKU) patient was received for *PAH* gene sequencing. One copy of a previously reported missense mutation, c.838G > A (p.E280K), was detected.[26] Since a second mutation was not detected, deletion/duplication analysis was ordered. A deletion was identified with four probes partially covering exon 6 and six probes covering a few hundred bases of immediately flanking intron 6 sequence (Figure 3a). Four probes over the 5’ end of exon 6 showed a normal hybridization signal. To confirm the partial deletion of exon 6, breakpoint PCR was conducted; an 801-bp deletion, with an insertion of 11 bp between the breakpoints in intron 6 and exon 6, was confirmed. The inserted bases corresponded to the reverse compliment of the intron 6 breakpoint (figure 3a bottom). The fragment analysis of the breakpoint PCR, and the complete sequence of the deletion locus is included in Additional file 3. A SINE (Short Interspersed Element) of the MIRb subfamily maps within the deleted sequence, 32 bases from the intron 6 breakpoint.

Subsequently, we detected one copy of the same *PAH* indel mutation allele, in a presumably unrelated, four-month-old who was diagnosed via newborn screening (NBS) to have elevated phenylalanine. *PAH* gene sequencing identified a c.168 + 1G > A splice donor site mutation (data not shown). Deletion/duplication analysis with aCGH detected intron6/exon6 deletion that was confirmed with the allele-specific PCR and sequencing developed for the previous patient.

### 267-bp deletion mutation encompassing exon 2 of the EMD gene

Emery-Dreifuss muscular dystrophy can be inherited in an autosomal recessive or an X-linked pattern, depending upon the gene that carries the mutation. Mutations in *EMD* cause X-linked Emery-Dreifuss muscular dystrophy [27]. We detected a 267-bp deletion encompassing exon 2 of the *EMD* gene in a 46-year-old male (Figure 3b). Despite the general criteria set for a minimum of four probes to determine a deletion call, two probes in a hemizygous condition were sufficient to prompt further investigation. Breakpoint mapping with allele-specific PCR revealed that certain probes within the deleted regions showed normal hybridization (Figure 3b). The fragment analysis of the breakpoint PCR, and the complete sequence of the deletion locus is included in Additional file 3.

### 12-bp intronic deletion in intron 5 of the BCKDHB gene

**Figure 4:**
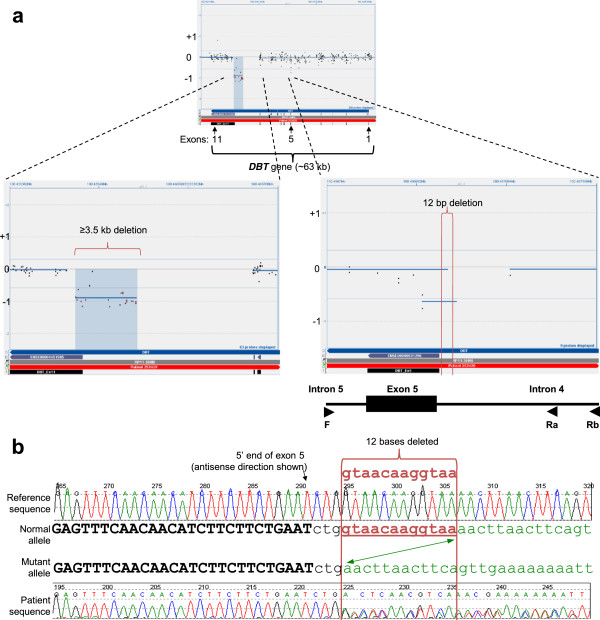
**Compound heterozygous deletions in the *DBT *gene.** Figure 4 shows CytoSure display of aCGH data with probes targeting the entire *DBT* gene, and zoomed-in views of two deletions: a large, >3.5-kb deletion encompassing the 5’ end of exon 11 is on the left, and a small 12-bp deletion in intron 4, three bases from the intron 4/exon 5 boundary, is on the right. The breakpoints of the small deletion are shown with vertical red lines. At the bottom is an illustration of the breakpoint PCR design, with the location of primers shown as arrows. Figure 4**b** shows the electropherogram indicating the deletion of 12 bases in one allele of the patient. The breakpoints are shown with two vertical red lines.

No mutation was detected by sequence analysis of the *BCKDHA*, *BCKDHB*, and *DBT* genes in a sample from a 13-year-old patient with a biochemical diagnosis of MSUD. However, aCGH analysis resulted in one CBS deletion call within the *BCKDHB* gene encompassing the 5’ end of exon 11. In addition, manual review picked up a possible small deletion at the 5’ end of exon 5 based on only two probes that map to the sense and antisense of the same 60-bp sequence. Breakpoint PCR and sequencing of amplicons across this region revealed a 12-bp deletion (Figure 4). This c.344-4del12 change is only three bp from the splice acceptor site at the 5’ end of exon 5 and was not detected in the original sequence analysis, since the original amplification primers hybridized to the deleted region, resulting in allelic dropout. The sequencing primer had to be placed very close to the splice site to avoid amplification through an intronic AT-rich simple repeat region distally. The fragment analysis of the breakpoint PCR, and the complete sequence of the deletion locus is included in Additional file 4.

### False deletion call due to an insertion mutation in the POMT1 gene

**Figure 5:**
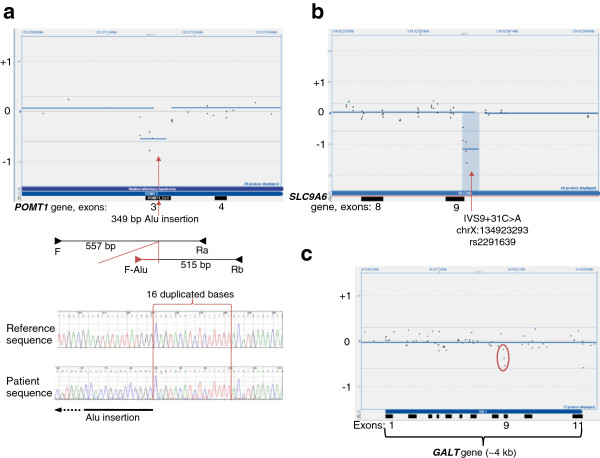
**False deletion calls. Figure**5 shows CytoSure display of three independent sets of aCGH data, where probe hybridizations on three separate genes resulted in false deletion calls. **5a**) Zoomed-in view of the *POMT1* gene*,* where red arrow marks the breakpoint in exon 3, where an Alu insertion interrupts probe hybridization. Below the CytoSure view is an illustration of the breakpoint PCR design, with the location of primers shown as arrows. At the bottom is an electropherogram of the allele with the Alu inserted. The 16 bases, duplicated in the two ends of the inserted element, are shown within two vertical red lines. **5b**) Zoomed-in view of the *SLC9A6* gene*,* where red arrow marks the location of a SNP in intron 9 that is targeted by all the probes that triggered the false deletion call. **5c**) The entire *GALT* gene, with three probes showing slightly poor hybridization due to compound heterozygous missense changes.

In some suspected intragenic deletions, further investigation leads to the discovery of an insertion, SNP, or point mutation causing poor hybridization. For example, sequence analysis of the *POMT1* gene identified a copy of a c.2167dupG mutation in exon 20 in a patient with Walker-Warburg syndrome (WWS) (data not shown) [28]. Finding one definite mutation triggered reflex deletion duplication testing. Analysis by aCGH showed four overlapping probes over exon 3, suggesting a possible deletion (Figure 5a). Breakpoint PCR and sequence analysis identified an Alu insertion (c.160_161ins349). The c.160_161ins349 Alu insertion has been previously reported in a patient with WWS in *cis* with a c.2203C > T nucleotide change in exon 20 that was also found in our patient [29]. By placing the forward primer within the Alu sequence, the presence of the Alu was confirmed. The fragment analysis of the breakpoint PCR is included in Additional file 5.

### False-positive deletion call due to hemizygous SNP in the SLC9A6 gene

A mutation in the X-linked *SLC9A6* gene in males results in intellectual disability, epilepsy, and ataxia, a phenotype similar to Angelman syndrome [30]. A possible deletion at the 5’ end of exon 9 of the *SLC9A6* gene in a three-year-old male patient was found to be a false-positive call. Since this is an X-linked gene, a true deletion call is expected to cross well below the threshold. The deletion call here did fall below the -1, but not to the degree expected in a hemizygous deletion (Figure 5b). Sequencing of exon 9 and flanking intronic sequences revealed a hemizygous single nucleotide polymorphism SNP (c.1140 + 31C > A; rs2291639). All probes suggesting a deletion encompass this SNP and result in poor hybridization on the aCGH. An electropherogram trace encompassing this SNP is included in Additional file 5.

### Low probe hybridization due to compound heterozygous missense changes in the GALT gene

A five-year-old patient with galactosemia was referred for *GALT* gene sequencing [31]. Sequence analysis of exon 9 of the *GALT* gene identified one copy of the c.855G > T (p.K285N) mutation and one copy of a c.844C > G (p.L282V) nucleotide change of unknown significance, in this individual. Parental testing showed that these two missense changes (a mutation and a variant of unknown significance) were in *trans*. Since the c.844C > G (p.L282V) nucleotide change has not been previously reported in a patient with galactosemia, aCGH was ordered to rule out the possibility of a deletion or duplication (Figure 5c). The three probes highlighted with a red circle overlap both the missense changes, demonstrating low hybridization that can be appreciated upon manual review. An electropherogram trace encompassing these nucleotide changes is included in Additional file 5.

## Discussion

Custom-designed high-density oligonucleotide arrays for molecular diagnostics are used to target specific disease-associated genes and are designed to detect single and multiple exon deletions and duplications [7,13,19,20]. The limit of detection in terms of the size of pathogenic deletions has improved immensely with the implementation of high-resolution, gene-targeted aCGH in diagnostic genetics [32,33]. The smallest size of deletion that analysis software can detect depends upon the density of probes targeting that sequence and the criteria set for software-generated calls. For example, if four consecutive probes targeted an overlapping sequence, and all four crossed the threshold set to detect deletions in our method (-0.6 log2 ratio), then a call could be generated even for deletions smaller than the length of the probes. As the size of a deletion gets closer to the limit of detection, the confidence in a call becomes weaker, and an alternate confirmation is necessary. Investigating suspicious events with breakpoint mapping helped us elucidate the true detection limit of our gene-targeted aCGH design. Several cases where software-generated calls did not cross the threshold nevertheless aroused suspicion upon manual review and warranted further investigation.

Detection of deletions is highly sensitive in the hemizygous genotype of males with X-linked disease. It is important to obtain relevant clinical information, family history, and any biochemical findings to help interpret the results of molecular testing; identification of a single copy of one mutation by gene sequencing for a patient suspected of an autosomal recessive disorder is also an indication to investigate any suspicious microarray data.

The smallest deletion we detected with aCGH was the 12-bp intronic deletion in the *DBT* gene of a child with a biochemical diagnosis of MSUD (Figure 4). The call made due to this deletion was only due to the hybridization of two probes targeting the same 60 bp and was only appreciated upon manual review. The location of these probes mapped to the sequence that the primer used in sequencing. Therefore, this deletion was not detected by sequencing due to allelic dropout, highlighting the fact that it was ultimately detected based on high clinical suspicion, the presence of one copy of another mutation in the same gene, and keen manual review.

Selection of probes and the density and redundancy in the coverage in the array design, are critical in the detection of intragenic CNVs. Not all probes perform equally well. Both deletions in the *STK11* gene described here failed to generate a call that crossed the threshold set for deletions (-0.6 log2 ratio). Several probes within the deletions had discrepant array and breakpoint PCR data (Figure 2 and 3). These data may indicate that the probes may be prone to non-specific signal and should be redesigned or removed. However, it is important to recognize the possibility that sequences within a deleted region may have translocated to another location within the gene, or elsewhere in the genome, and consequently may carry the potential for clinical implications. However in the *STK11* deletions presented, the clinical findings are consistent with the deletions alone.

Most deletions reported here had microhomology of at least a few bases at the breakpoints. This is consistent with the replication-based mechanism and break-induced repair (BIR) mechanism hypothesis for such events [4,34,35]. Interestingly, in the 801-bp deletion encompassing part of exon 6 of the *PAH* gene, there was an 11-bp insertion that corresponds to the reverse compliment of bases along the intron 6 breakpoint, demonstrating the involvement of at least two double-strand breaks in the mechanism resulting in this deletion (Figure 3). The familial *HPRT1* deletion also had an insertion of 69 bp (Figure 1). In this mutation, the inserted bases aligned to a region on chromosome 5 (chr5p13.1:40,844,202-40,844,270/hg18) that has no homology to the locus on the X-linked gene. A second recurrent theme at breakpoints is close proximity of SINEs (Short INterspersed Elements) or other repeat tracks, suggestive of non-allelic homologous recombination.

In spite of the fear that higher probe density generates more noise in aCGH data, in our experience, the greater the number of probes within a deleted area generally helped in its detection. This is true even when there is redundancy in the probes; for example, the *EMD* gene deletion, where the same 60 bp were targeted by probes complimentary to the two strands. There was a definite call made by CBS software for the 801-bp *PAH* gene deletion. In contrast, the larger *STK11* deletions did not cross the threshold for the software to generate a call. This difference is due entirely to probe density, which is higher for the *PAH* gene in our array. With sufficient data, probe performance can be evaluated and array design modified for optimal sensitivity. Possible deletions that were deemed false positive did demonstrate how single nucleotide changes could decrease the hybridization of probes, highlighting the sensitivity of this technology (Figure 5). In one case, the call generated did lead to the identification of a pathogenic Alu insertion. We have found that familiarity with specific probe performance within a gene helps differentiate between informative variation and noise.

It is important to remember that oligonucleotide arrays have the same limitations as any method that relies on hybridization to unique sequence probes. Therefore, repeat sequences are not targeted, and pseudogenes and homologs will interfere with assessments. Also, the information on copy number variation gives no insight on the orientation or location of insertions, duplications or rearrangements.

Gene targeted aCGH technology described here is complementary to diagnostic analyses utilizing next generation sequencing (NGS) that have been rapidly adopted in clinical laboratories, especially for genetically heterogenous diseases where more than one gene can contribute to a disorder. Several gene panels are being offered by clinical laboratories, for example gene panels for X linked intellectual disability, cardiomyopathy, neuromuscular disorders and congenital disorders of glysosylation. Detection of small indels from NGS data is still not optimal, and detection of CNVs via NGS cannot be easily adopted in clinical laboratories since the required lowered stringency would introduce a high false positive variant call rate. Gene targeted can easily fill these gaps and make the gene panels complete by offering combination of NGS based test and gene targeted arrays to detect the near complete range of mutations detected in genes.

## Conclusion

We present the examples of pathogenic intragenic deletions ranging from several kilobases to as small as 12 bases, to highlight the limit of detection with high-density gene-targeted aCGH. Although probe coverage and performance are critical parameters to consider, however, there is not a minimum criteria of probe density that can be applied across all genomic sequences of interest. Based on our experiences, rigorous efforts to detect the smallest of these intragenic CNVs extend beyond simple aCGH analysis algorithms. As the size of deletion gets smaller, the cumulative data from all encompassing probes is insufficient to make a confident call. CBS software does allow identification of these events during manual review, even when the call does not cross the threshold set for the detection. For detection of these smaller CNVs, we routinely investigate further if one or more of the following criteria are met: a) the call was generated with at least two entirely non-overlapping probes, b) the location of the call overlaps with a primer used in sequencing that may have caused allelic dropout, c) the disease gene is recessive, with one mutation within the gene identified, or d) the disease gene is dominant, with a strong clinical suspicion in the patient. Ultimately, these data can be used to track individual probe performance across samples to improve the sensitivity of the array. In conclusion, high-density targeted aCGH is a very powerful tool for detection of intragenic deletions, and the identification of novel intragenic deletions and duplications will help expand the known spectrum of disease-associated genes.

## Methods

### Array design

All array data discussed in this manuscript were generated using the custom-designed EGL_NMD_NBSplus_v1 array. This 4X180K array was developed on the Agilent Technologies (Santa Clara, CA) aCGH platform using the Genefficiency service (Oxford Gene Technology (OGT), Yarnton, Oxford OX5 1PF UK). OGT uses proprietary ink-jet *in situ* printer technology (IJISS) developed by Rosetta InPharmatics (Kirkland, WA) and Agilent Technologies that allows *in situ* synthesis of long oligonucleotides. The probes are ~60 bp in length and annotated against NCBI build 36.1 (UCSC hg18, March 2006). This array has 207 control probes and 15,028 backbone probes spread in between regions of interest. 157,448 probes are targeting 261 genes (see Additional file 6).

### aCGH protocol

DNA was extracted from whole blood collected in EDTA (purple-top) collection tubes and from amniocytes received for prenatal testing using the Puregene DNA extraction kit (Qiagen, Valencia, CA) according to the manufacturer’s recommendation. aCGH was performed following the manufacturer’s protocol (Agilent Technologies, Santa Clara, CA). Each patient’s DNA was spiked with a combination of PCR products (spike-in) unique to each sample per array. The reference DNA was used from two pools (male and female) from normal individuals, run as a same-sex control. DNA was sonicated using a Branson Sonifier 450 with cup horn (Danbury, CT) and visualized on a two-percent agarose gel prior to labeling, as a quality control measure. Each patient and reference DNA was labeled with Cy3 and Cy5 9mer primers, respectively. Purification of labeled products, hybridization, and post-wash of the array was carried out according to Agilent’s recommendation and with their proprietary solutions. Array slides were scanned with Agilent’s High-Resolution C Scanner and extraction software.

### aCGH analysis

CytoSure Interpret software 02002 (OGT) was used for analysis of array data (referred to as CytoSure). The program uses the Circular Binary Segmentation (CBS) algorithm to generate segments along the chromosomes that have similar copy number relative to reference chromosome [21]. Averaging of the segments is with median value of all segments on a chromosome as the baseline. Deletion or duplication calls are made using the log2 ratio of each segment that has a minimum of four probes. Threshold factor for deletions was set as a log2 ratio of -0.6 that is less stringent than the theoretical log2 score of -1 (heterozygous deletion log2(1/2) = -1; No change in allele number log2(2/2) = 0; heterozygous duplication log2(3/2) = 0.59). The software uses the standard deviation of the log2 ratio to calculate a deviation log ratio (DLR), which is used as a quality control check. A DLR of 0.08-0.19 is accepted, 0.20-0.29 is borderline, and ≥0.30 is rejected. The DLR for all arrays shown was scored by this scale. Data is analyzed only for the gene ordered for testing. The data for others genes is masked and not analyzed.

### Breakpoint mapping design

CytoSure segment calls were used to generate minimum and maximum genomic coordinates of possible aberrations using NCBI build 36.1 (UCSC hg18, March 2006). The UCSC Genome Browser was used to determine the composition of the involved DNA [36,37]. We assessed repeat tracks and segmental duplications, as well as all annotated SNPs [24,38-43]. Breakpoint mapping by PCR was used to confirm deletion calls encompassing all or part of at least one exon. Primers for breakpoint PCR were designed using Light Scanner Primer Design software (Idaho Technologies Inc, Salt Lake City, UT). Several primer sets were designed by walking along the DNA sequence proximal and distal to the possible CNV.

### PCR

All primers were ordered from Integrated DNA Technologies, Inc. (Coralville, IA). In all PCRs, 50 ng of genomic DNA was amplified in a 50-μl reaction that had a final composition of 2 U FastStart *Taq,* 1X FastStart *Taq* buffer with MgCl_2_, and 0.2 mmol/L dNTPs (Roche Applied Science, Indianapolis, IN), as well as 10 pmol forward and reverse primers (for primers sequences see Table 2). The PCR cycling had an initial melting at 95°C for 3 min followed by 40 three-temperature cycles (60 s at 95°C, 60 s at the lower of the two primer Tms, and 72°C for 1 min). The 40 cycles were followed by a final extension at 72°C for 7 min, and then held at 4°C. 15 μl of each PCR product was visualized on a 2% agarose gel (Fisher Scientific, Waltham, MA). Primers that successfully amplified across the breakpoints for cases described are listed in table 1.

**Table 2:** Table lists primers used in confirming breakpoint mapping for the cases listed in this manuscript

**Primer name**	**Primer sequence**
PAH_bkpt_ex6_F	GCTAAATTAACATCCTCTTGACAGAA
PAH_bkpt_ex6_R	ACCCTTTCATGTGGGAAATC
STK11_bkpt_ex8_Fa	CCAGTGGCCTTGGGAGAA
STK11_bkpt_ex8_Fb	GAGATGCGCCAGGAAGG
STK11_bkpt_ex8_R	GGCTGGCTGCCAATGTG
STK11_bkpt_ex3_F	GTTGTGGGCCATTTTGGT
STK11_bkpt_ex3_Ra	TGGCCTCACGGAAAGGA
STK11_bkpt_ex3_Rb	CAGCAAAGATGGAGGCG
STK11_bkpt_ex3_Rc	ATTTTCCTGTGGGCCACAGG
STK11_bkpt_ex3_Rd	AATCAGCTGACAGAAGT
HPRT1_bkpt_ex5_Fa	TATATGACAGAGTATGATGAGAGCTACA
HPRT1_bkpt_ex5_Fb	GCCTCATTCTTATAACTAGCATAAGAAC
HPRT1_bkpt_ex5_R	ACAGTGGCTCATGCCTAT
EMD_bkpt_ex2_F	CTCGGCCGGTTTTGGTA
EMD_bkpt_ex2_Ra	CAGACTTCCCTCCCCTTTCT
EMD_bkpt_ex2_Rb	AGGTCTCAGGTCCTCCCTGT
DBT_bkpt_int4_F	CAGAGATACAAATGTACACTTCCTA
DBT_bkpt_int4_Ra	AGTTGTGTTTTCCTATTCTGAAGTAGTT
DBT_bkpt_int4_Rb	TGACATATCCACCAGGTACTAATAATTAAA
POMT1_bkpt_ex3_F	CAGGATTAGCCTTGCGTC
POMT1_bkpt_ex3_Ra	GGCAAGCAATAAACAAGATGC
POMT1_bkpt_ex3_Rb	TCTGTGGGACTAGGTATGAAAGG
F-Alu_sense	GTCTCGATCTCCTGACCTCG
SLC9A6_ex9_F	TCTACTGTGAAGAAAGAACCTCAG
SLC9A6_ex9_R	GGAAGAGGAGCCAAAATGAG
GALT_ex9_F	CTAGGCACTGGATGGAGGTT
GALT_ex9_R	TCACTAGGCTGAGCCCCAGG

### Sequencing

PCR products were purified using the Millipore Ultrafiltration PCR purification kit (Millipore, Billerica, MA). Sequencing reactions (15 μl total) were prepared with the BD v3.1 sequencing kit (Applied Biosystems, Foster City, CA). Each PCR product was sequenced bidirectionally using the amplification primers. The sequencing reaction products were purified using a Sephadex cleanup plate (Edge Biosystems, Gaithersburg, MD) according to the manufacturer’s instructions. Products were heat-denatured (5 min at 95°C) and sequenced on a 3730xl capillary sequencer (Applied Biosystems, Carlsbad, CA). Sequence analysis was performed using Mutation Surveyor v2.61 software (SoftGenetics, State College, PA).

## Abbreviations

PCR: Polymerase chain reaction; aCGH: array Comparative Genomic Hybridization; MLPA: Multiple ligation-dependent probe amplification; CBS: Circular binary segmentation; CNV: Copy number variation; SNP: Single nucleotide polymorphism; SINE: Short interspersed element; NHEJ: Non-homologous end-joining; MMBIR: Microhomology-mediated break-induced replication; DMD: Duchenne muscular dystrophy; MSUD: Maple syrup urine disease; LNS: Lesch-nyhan syndrome; HPRT: Hypoxanthine guanine phospho-ribosyl-transferase; PJS: Peutz-jeghers syndrome; PKU: Phenylketonuria; EMD: Emery-dreifuss muscular dystrophy; WWS: Walker-warburg syndrome.

## Competing interests

All work presented was performed at Emory Genetics Laboratory. Since the completion of the work, author SHA has been employed by Medical Neurogenetics Inc, and author EC has been employed by Oxford Gene Technology. All other authors declare no conflict of interest.

## Author’s contributions

SHA designed and carried out the breakpoint mapping for confirmation, and drafted the manuscript. Targeted aCGH was developed, validated and implemented under the leadership of MH and EC. All authors were involved with aCGH data review. All authors read and approved the final manuscript.

## Authors’ information

This work was part of SHA’s project assignment during ABMG (American Board of Medical Genetics) fellowship at Emory University, under the guidance of MH.

## Supplementary Material

Additional file 1 **2319-bp deletion in the *HPRT1 *** **gene.** Click here for file

Additional file 2 **1325-bp deletion in the *STK11 *** **gene.** Click here for file

Additional file 3 **801-bp deletion in the *PAH *** **gene and 267-bp deletion in the *EMD *** **gene.** Click here for file

Additional file 4 **12-bp deletion in the *DBT *** **gene.** Click here for file

Additional file 5 **False deletion calls in the *POMT1*, *SLC9A6 *** **and *GALT *** **genes.** Click here for file

Additional file 6 **List of genes included in the custom-designed gene-targeted array.** Click here for file
