# Supplementary material for: Detection limit of intragenic deletions with targeted array comparative genomic hybridization
Source: BMC Genet. 2013 Dec 5;14:116. doi: 10.1186/1471-2156-14-116 (PMC4235222; doi:10.1186/1471-2156-14-116)
Supplement: Additional file 1 — 2319-bp deletion in the HPRT1 gene. [file 1471-2156-14-116-S1.pdf]

## Additional File 1

### Figure A

**Familial 2319-bp deletion encompassing exon 5 of the *HPRT1* gene.**
Cytosure displays of aCGH data from proband, carrier sister, and unaffected nephew are shown. Red ovals demarcate the probes that target the deleted locus.

### Figure B

**Fragment analysis of breakpoint PCR of the familial 2319-bp deletion encompassing exon 5 of the *HPRT1* gene.**
Figure shows an agarose gel with the deleted allele preferentially amplified in the proband and carrier sibling, while the normal allele did not amplify under the PCR conditions. Size ladder (Invitrogen 1 KB plus) is in the lane labeled as 1 kb+, while wild-type DNA in lane 1, proband DNA in lane 2, carrier sister's DNA in lane 3, and water in lane 4. The two alternative forward primers used in amplification are labeled as Fa and Fb. The expected sizes of fragments generated from normal wild type alleles are written on top of the gel and the sizes of the amplicons generated from deleted alleles are highlighted with an arrow on the right. Asterisks highlight the bands sequenced.

### Figure C

**Sequence from the deletion locus of the familial 2319-bp deletion encompassing exon 5 of the *HPRT1* gene.**
Sequence shows the breakpoints in relation to the adjoining sequence; repeat elements, SNPs, exons, and primers. Sequence coordinates are listed according to UCSC hg 18 build (March 2006), and the two interruptions demarcate the breakpoints. Nucleotides in bold and capitalized font represent exons. Nucleotides in bold and underlined font correspond to the primers used in breakpoint PCR. RepeatMasker are highlighted with blue font, SNPs (130 build) are highlighted in red font, and microhomology at breakpoints is shown boxed.

## Additional File 1, Figure A

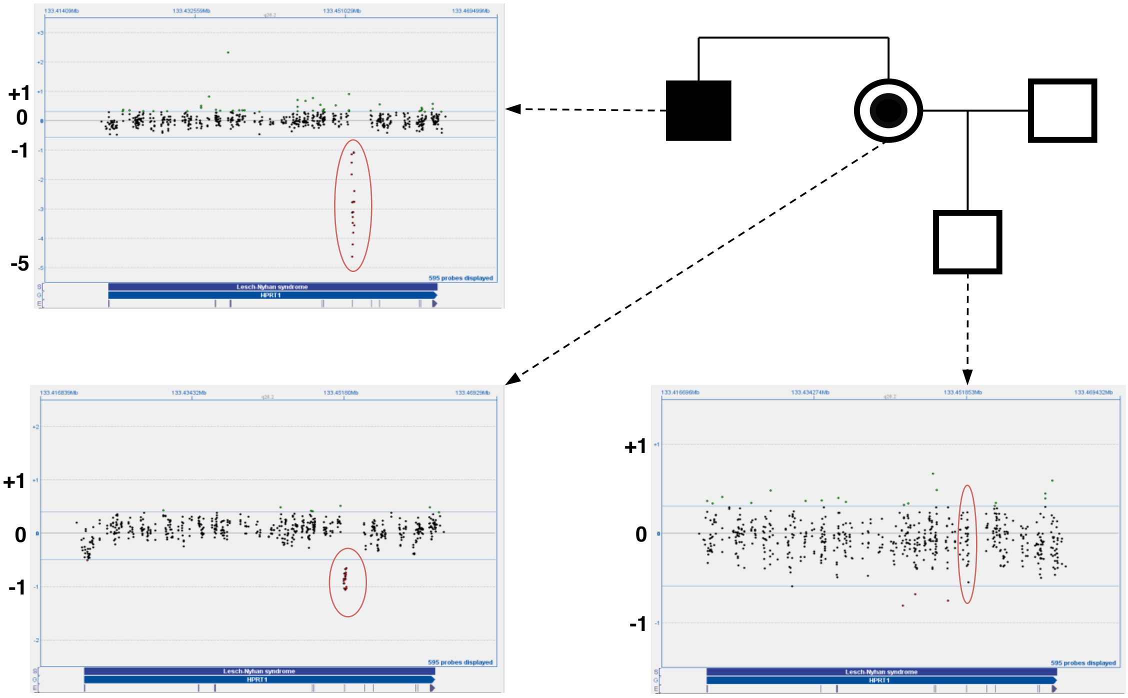


## Additional File 1, Figure B

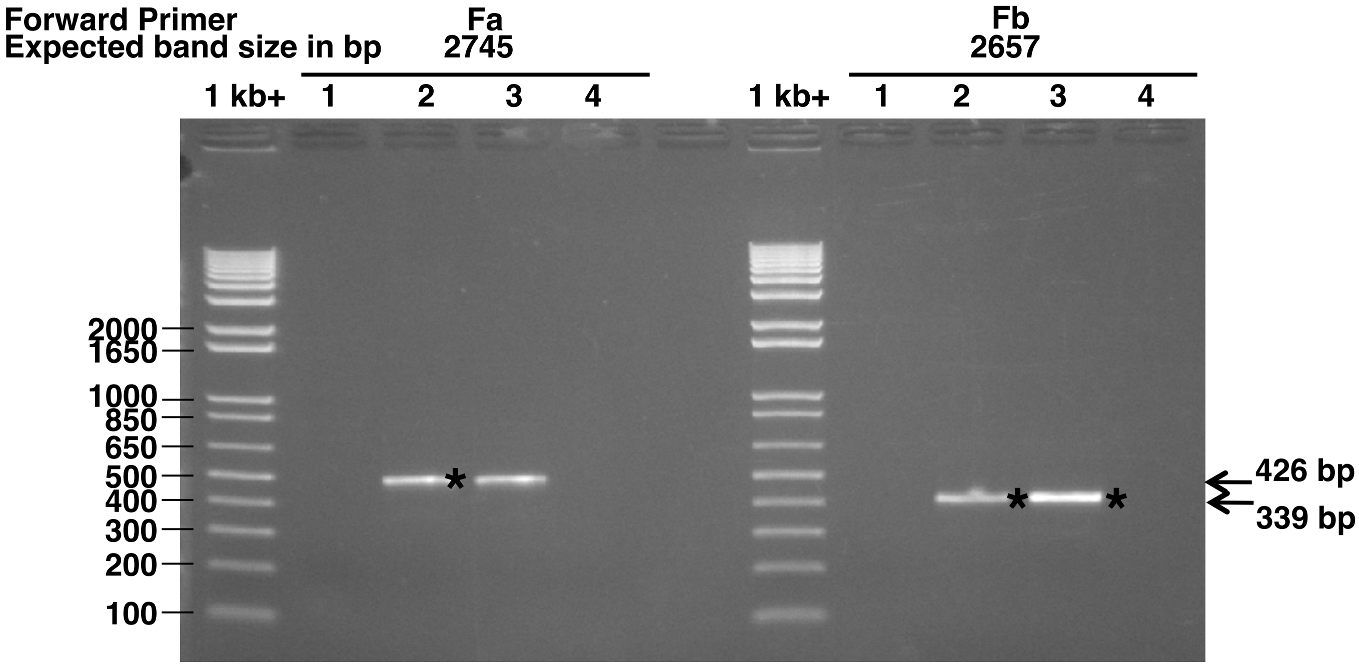

**Additional File 1, Figure C**

**chrX:133,451,101-133,453,900**
atatatagaacattaaaatcttaatgctatagt**tatatgacagagtatga** **Fa**
**tgagagctaca**gataaacaacacatcatgaatcttcttgtggcagtgttt
ataaccattatgtgaaatgct**gcctcattcttataactagcataagaac**a **Fb**
gataggactttctcgattttgaggggtaattattagatggtattttctgt
taaggactcttccagctataaaattcttaaatgtagaaagcgaagtgagg
gtttatggtgagaggaagcattggtatcatgttttagtgtagtccaagaa
ta
**(Breakpoint @ chrX:133,451,403 in intron 4)**
tggacacatccagaaaatgcagatcaagtttagcctaatgagaaaata
tattttggagtccatatggtaaattaaattatgtgatttttgagttattg
tacaaatataattcttagaatgttagagtcaggagactataagagaccaa **MIR3**
ctgcttcaagtttcatttaacacatgggaaactaaggc**c**agagaaatttc
aagacttgcccaagattagacctcttgttaagtaatgaaagtgttttaaa
aacaggtgggtcaaattctgtttttaaaatttccattatgatgaaaattt
cagtattacaggcttccaaatcccagcagatgggccacttgtttaaagga
gagtttgatataataaagcatctaaaaacaagagtttggataattcctta
gggttgttatgatgtgatttgacttataattggaaataccgttttattca
ttgtactgattttcatttctctttttcttctag**AATGTCTTGATTGTGGA** **Exon 5**
**A**gtaagttcacatttacttttaatataacatttatgacttttctaactta
gtatgcaccatcctaaaggtaagccagggagagaaattcctctgcatcag
tttta-----------------1390 bp----------------tcttc
aattcacttggaccttttgcatttaattttaattttatttttttttaaga
cagagtctcactctgtcaccaggctggagtgcagtggtatgatctcagct **Alu FLAM_C**
cactaactactctgcctcccaggttcaagcaattctcatgtctcagcctc
ccaagtagctgggactacaggtgtgcgccaccatgcctggctaattgtgt
gtgtgtgtgtgtgtatgtatgtatgtatatatgtgtgtgtgtgtatatat **TA-rich repeat**
atatatacaca**c**aca**t**a**t**ataaatatatatacatatatatatatacacac
atatataaatatatatacata
**(Breakpoint @ chrX:133,453,721 in intron 4)**
tatatatatacacacacacacacatatat
atatatatagttttttttttttttaagtagagatgggttttgccatgtt
ggccaggctggtctggcctcaagccatcctcccacctcggcctcgcaaag
tgctggtatt**ataggcatgagccactgt**gcctggcctgcatttcatttta **R**
