# Supplementary material for: Detection limit of intragenic deletions with targeted array comparative genomic hybridization
Source: BMC Genet. 2013 Dec 5;14:116. doi: 10.1186/1471-2156-14-116 (PMC4235222; doi:10.1186/1471-2156-14-116)
Supplement: Additional file 2 — 1325-bp deletion in the STK11 gene. [file 1471-2156-14-116-S2.pdf]

Additional File 2

Figure A. **Fragment analysis of breakpoint PCR of the familial 1325-bp deletion encompassing exon 8 of the *STK11* gene.**
Figure shows an agarose gel with the deleted allele preferentially amplified in the proband. Size ladder (Invitrogen 1 KB plus) is in the lane labeled as 1 kb+, while wild-type DNA in lane 1, proband DNA in lane 2, and water in lane 3. The two alternative forward primers used in amplification are labeled as Fa and Fb. The expected sizes of fragments generated from normal wild type alleles are written on top of the gel and the sizes of the amplicons generated from deleted alleles are highlighted with an arrow on the right. Asterisks highlight the bands sequenced.

Figure B. **Sequence from the deletion locus of the the familial 1325-bp deletion encompassing exon 8 of the *STK11* gene.**
Sequence shows the breakpoints in relation to the adjoining sequence; repeat elements, SNPs, exons, and primers. Sequence coordinates are listed according to UCSC hg 18 build (March 2006), and the two interruptions demarcate the breakpoints. Nucleotides in bold and capitalized font represent exons. Nucleotides in bold and underlined font correspond to the primers used in breakpoint PCR. SNPs (130 build) are highlighted in red font, and microhomology at breakpoints is shown boxed.

Figure C. **Fragment analysis of breakpoint PCR of the familial 971-bp deletion encompassing exon 3 of the *STK11* gene.**
Figure shows an agarose gel with the deleted allele preferentially amplified in the proband with one of four reverse primers used. Size ladder (Invitrogen 1 KB plus) is in the lane labeled as 1 kb+, while wild-type DNA in lane 1, proband DNA in lane 2, and water in lane 3. The four alternative reverse primers used in amplification are labeled as Ra, Rb, Rc and Rd. The expected sizes of fragments generated from normal wild type alleles are written on top of the gel. The size of the amplicon generated from deleted allele, when amplified using Rd primer, is highlighted with an arrow on the right. Asterisks highlight the bands sequenced.

Figure D. **Sequence from the deletion locus of the the familial 97-bp deletion encompassing exon 3 of the *STK11* gene.**
Sequence shows the breakpoints in relation to the adjoining sequence; repeat elements, SNPs, exons, and primers. Sequence coordinates are listed according to UCSC hg 18 build (March 2006), and the two interruptions demarcate the breakpoints. Nucleotides in bold and capitalized font represent exons. Nucleotides in bold and underlined font correspond to the primers used in breakpoint PCR. RepeatMasker are highlighted with blue font, SNPs (130 build) are highlighted in red font, and microhomology at breakpoints is shown boxed.

Additional File 2, Figure A

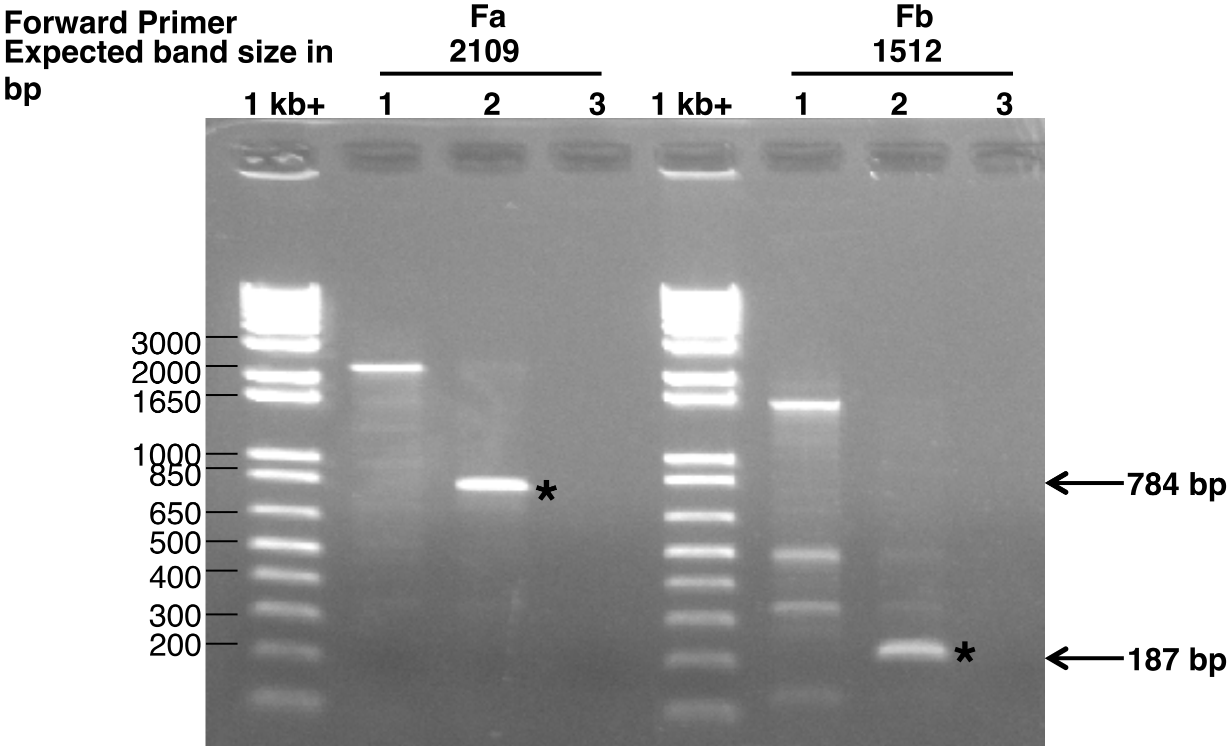

Additional File 2, Figure B

```
chr19:1173251-1174850
aggcatggagatgcgccaggaagggcacagctggtcccaaacactggcga Fb
gagcctctcttttcccctcctcctggggctcccagcagcagggtgtggc
tgggatccagcccagggcccccagctccatgacagggaagacagagcagc
Ggacggggtcagcag
(Breakpoint @ chr19:1,173,416 in intron 7)
                     gccccacagtgccgcctccctcacttcgtgggctc
tgctcctctgcaccagcccctggaggcccttgagccgtctgctggagccc
ctccgagccccgaggccacccactgagaccggctctgggagtgggagtgt
ccggacccctgaggcgctggtgctgattgtgccttgggggtctctgcaca
gctcgggtcatctgggcgcctggcggggactggggctgcccccgatagc
ctcctgggctgggatgtgctcagggcccccagacccccttctggccttt
gctggctttgcagccagcatccatctggtgggtgctggcttctgagtgcc
acctgggacacaggcctcagggtggagggacatctgtcaggcttggagt
caggtcagcctgcctgctcctagaggacatggctgagcttctgtggtcac
agccaccccttgcacggcctggtcccagctcctgagtgtgtggcaggtac
cctgggcccagaggagctgggtcggaaaactggaccgccctggtgccagc
ctgacaggcgccactgcttctgggcgtttgcagCTGGTTCCGGAAGAAAC
ATCCTCCGGCTGAAGCACCAGTGCCCATCCCACCGAGCCCAGACACCAAG Exon 8
GACCGGTGGCGCAGCATGACTGTGGTGCCGTACTTGGAGGACCTGCACGG
CGCGGACGAGGACGAGGACCTCTTCGACATCGAGGATGACATCATCTACA
CTCAGGACTTCACGGTGCCCGgtgagtctggcgggggcccctgcccggct
ctgctgactcggccaggatgtcccacgggagcagggtgcctgcctgtctg
caaca------------390 bp not shown------------aggcg
tctgaggcagggcttagagcggagcgcggcttgcagaagagcgagggctc
Agacctttcaggagaggaagcctctcggccggcgccgcag
(Breakpoint @ chr19:1,174,740 in intron 8)
                                          tagtgcctga
ggaggagctcagggccttagcgtaggggcggcccacattggcagccagcc R
cctccccgccatgctcccggcttggctgtgttcggcccagggctgggccg
```

Additional File 2, Figure C

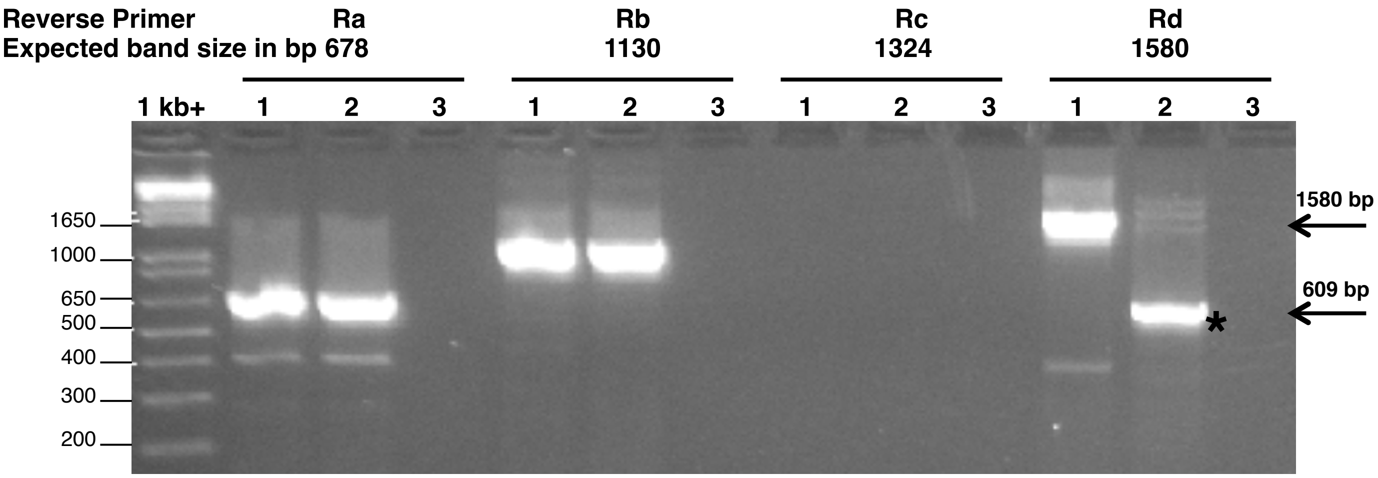

Additional File 2, Figure D

chr19:1,169,801-1,171,400

cgggctgacc**gttgtgggccattttggt**cgtggctgggcgtgtcctcgtg **F**
tcatctgtggacacccccatgggtcttacgggcacagcctccctacgggg
actttgcttcctaaggccctgtgcccagagcaagagccagaagtggtcct
gaggctggggctgtgttccctgagccacgcggtcaggggccctgggaccg
tcctgcatgggcccgagcctgcttgggggggcgtccaggaggcaccatcc
cccgcccatgggcagggtggg
(Breakpoint @ chr19:1,170,072 in intron 2)
ggacgtgagccccgcaggaacgctgcccc
aagagtcagccctgtcctccccttccccgtaggctccttcctcctgggac
gctggggcccctgggccttttcagaggggtggctgagggcagggtgggcc
ctggtcccgaggaggggcaaggtgggtgcagagggtccctccagagcccc
ttttctggccccgtgctccctgggcctgtgagtggggccgccccctgag
ctgtgtgtccttagcgccccac**GTATATGGTGATGGAGTACTGCGTGTGT**
GGCATGCAGGAAATGCTGGACAGCGTGCCGGAGAAGCGTTTCCCAGTGTG **Exon 3**
CCAGGCCCACGGgtgcgtgcgcggggcaggggccagggtggggcggggc
cgggggccaggcagggcaggc**tcctttccgtgaggcca**cactgcttgtcc **Ra**
tgatattcattgacatgaaggcccaagtttttttgttttttttgttttttt
gtgtttttttttcgagatggagtctcactctgtcgcccaggctggagtgca
atggtgcgatctcggctcactgcaagctccgcctccgaggttcacgccat **AluY**
tcttctgcctcagcttcccgagtagctgggattacaggcgcccgccacca
cgcccggctaatttttttgtatttttagtagagacggggtttcaccgtgtt
agccaggatggtctcaaactcctgacctcgtgatccgcctgcctcagcct
cccaaagtgctgggattacaggcatgagctaccacgcccggccttgtaaa
ggcccaagttttttaaaaacagttttggggtcccccatgtgtggcatccac
aggcagggctgctgccaacctcc**cgcctccatctttgctg**ggcctgctgc **Rb**
ctgaggccagtggcctgcttccagcccatcgctggcagccgcctgccctg
accagatctcctggatgcaggtctgtggcctcagagtcaggg
(Breakpoint @ chr19:1171042 in intron 3)
ccccttgc
tgctgcaggaccacaggggcagggaggggcctgctgttccagcaagactt
tggggtgcagccgg**cctgtggcccacaggaaaat**gagacctgtggacatc **Rc**
cggggccctgccagacgtggctcggccggacgagggtggccactgcaggc
gcaggtgtggctccctgctggacctagcctttcctctgtcctgtgtgcct
ggacttctgtgacttcccagctgggcctgtggtgtttgggaggctcccag
gcagctgcaaaggggacccctgtgaggggcagggaggcctcggccccagg
acgggtgtgtgctgcccgcag**GTACTTCTGTCAGCTGATTGACGGCCTGG** **Rd**
