# Supplementary material for: Detection limit of intragenic deletions with targeted array comparative genomic hybridization
Source: BMC Genet. 2013 Dec 5;14:116. doi: 10.1186/1471-2156-14-116 (PMC4235222; doi:10.1186/1471-2156-14-116)
Supplement: Additional file 3 — 801-bp deletion in the PAH gene and 267-bp deletion in the EMD gene. [file 1471-2156-14-116-S3.pdf]

Additional File 3

Figure A

Fragment analysis of breakpoint PCR of the familial 801-bp deletion encompassing part of exon 6 of the *PAH* gene.

Figure shows an agarose gel with the deleted allele amplified in the proband. Size ladder (Invitrogen 1 KB plus) is in the lane labeled as 1 kb+, while wild-type DNA in lane 1, proband DNA in lane 2, and water in lane 3. The expected size of fragment generated from normal wild type allele is 1134 bp. The size of the amplicon generated from deleted allele is 344 bp. Asterisks highlight the bands sequenced.

Figure B

Sequence from the deletion locus of the familial 801-bp deletion encompassing part of exon 6 of the *PAH* gene.

Sequence shows the breakpoints in relation to the adjoining sequence; repeat elements, SNPs, exons, and primers. Sequence coordinates are listed according to UCSC hg 18 build (March 2006), and the two interruptions demarcate the breakpoints. Nucleotides in bold and capitalized font represent exons. Nucleotides in bold and underlined font correspond to the primers used in breakpoint PCR. RepeatMasker are highlighted with blue font, SNPs (130 build) are highlighted in red font, and microhomology at breakpoints is shown boxed.

Figure C

Fragment analysis of breakpoint PCR of the familial 267-bp deletion encompassing part of exon 1 and all of exon 2 of the *EMD* gene.

Figure shows an agarose gel with the deleted allele preferentially amplified in the proband. Size ladder (Invitrogen 1 KB plus) is in the lane labeled as 1 kb+, while wild-type DNA in lane 1, proband DNA in lane 2, and water in lane 3. The two alternative reverse primers used in amplification are labeled as Ra and Rb. The expected sizes of fragments generated from normal wild type alleles are written on top of the gel and the sizes of the amplicons generated from deleted alleles are highlighted with an arrow on the left. Asterisks highlight the bands sequenced.

Figure D

Sequence from the deletion locus of the the familial 267-bp deletion encompassing part of exon 1 and all of exon 2 of the *EMD* gene.

Sequence shows the breakpoints in relation to the adjoining sequence; repeat elements, SNPs, exons, and primers. Sequence coordinates are listed according to UCSC hg 18 build (March 2006), and the two interruptions demarcate the breakpoints. Nucleotides in bold and capitalized font represent exons. Nucleotides that are underlined correspond to the primers used in breakpoint PCR. RepeatMasker are highlighted with blue font, SNPs (130 build) are highlighted in red font, and microhomology at breakpoints is shown boxed.

Additional File 3, Figure A

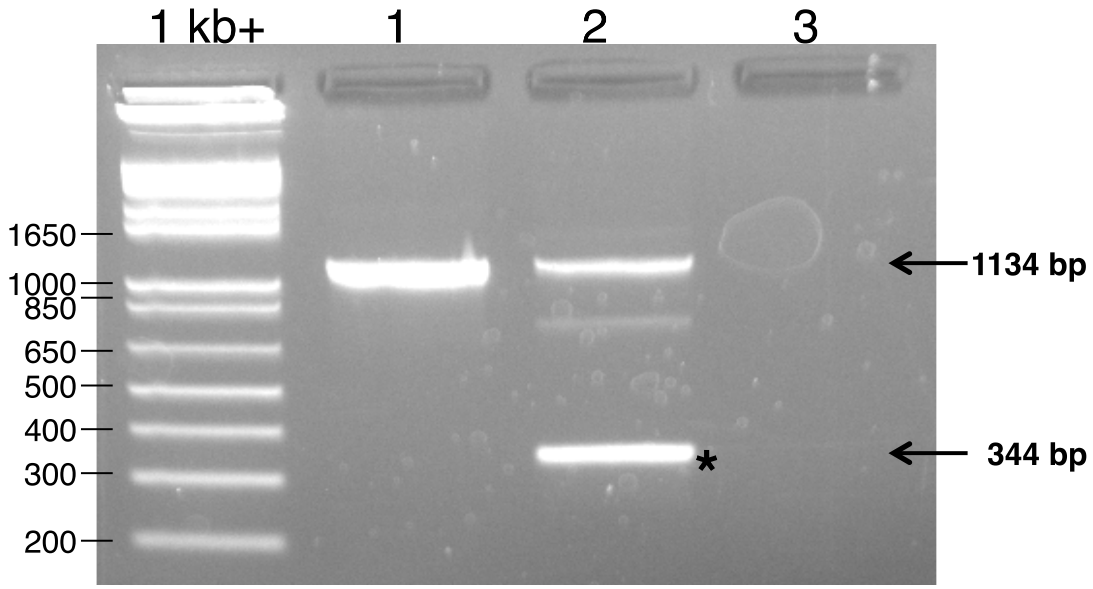

Additional File 3, Figure B

chr12:101,772,301-101,773,450
ttgtttttt**gctaaattaacatcctcttgacagaa**agagtaacacccattt
Cacctggatatactgtacagagaggcttgcgactcaatgtacttacct **F**
(Breakpoint @ chr12:101,772,399 in intron 6)
at
t**a**agctactcagatttcaattcagtccaaa**tt**cactgaatacctgctgta
tatgcaagactgtgctggttgtttccatatgcagcaccttacttcattct
cataatttccctatgggaaaagctaaagagttgatacagtgtccatttga **MIRb**
cagatgacaaaactgaggcaggtttactcagctggagaggat**t**gaaggca
ggattcataccagatgcacagactcagagctcagggctcttggcaccatc
cccgaaaatagcacatttcttacacaaacacacactcctaactcataaca
cagcaggaactacagggcaaacaaaacaaaacaaaacaaaa**a**aaaacctc
agtgaagcaccttgggctttaagtgtgaaagaaaatacttttcagggaca
ggtacacggcaaaatccacagcctcaggtgtttgattgaatgaaagtgga
taaacacagtaggggctggagggaaggcagagcacagtgaaatttagttc
ttcctggaggaatcaacctgcatgcattcctacaagcacatgctttcata
cttgcctccacatacttgtcttccccttccctctcctctgcctcaatcct
cccccaactttctgcagggccattga**c**cctgatgtggacttac**TCTGCAG**
GAACTGAGAAACGTCTTCCAGCTGGGGAATGTTATCTTCATGGAAGCCAC **Exon 6**
AGTACTTTTCAAGAAGTGGAAAAATGTGATTGTACTCATAGCAAGCATGG
GTTTTATACAAGGACTTCAGAGTCTTGAACACTGTGCCCCATGTTTTCT
(Breakpoint @ chr12:101,773,199 in exon 6)
T
TTCTTCCTCCATGTATTCCACTCGAGGGATGGGCTGCCCActaga**a**taca
ggcacaaaataggtgtctcaagcagggcaggggcacagcagaa**c**gcaggt
taggttagcagagggagtcggggaccagaacctgtgagctgccatcactt
gctacagtgaatttcatacattatttgaaagagaaaaattccacatcttg **R**
atataggaaacattgttgtttt**gatttcccacatgaaagggt**gatttggt

Additional File 3, Figure C

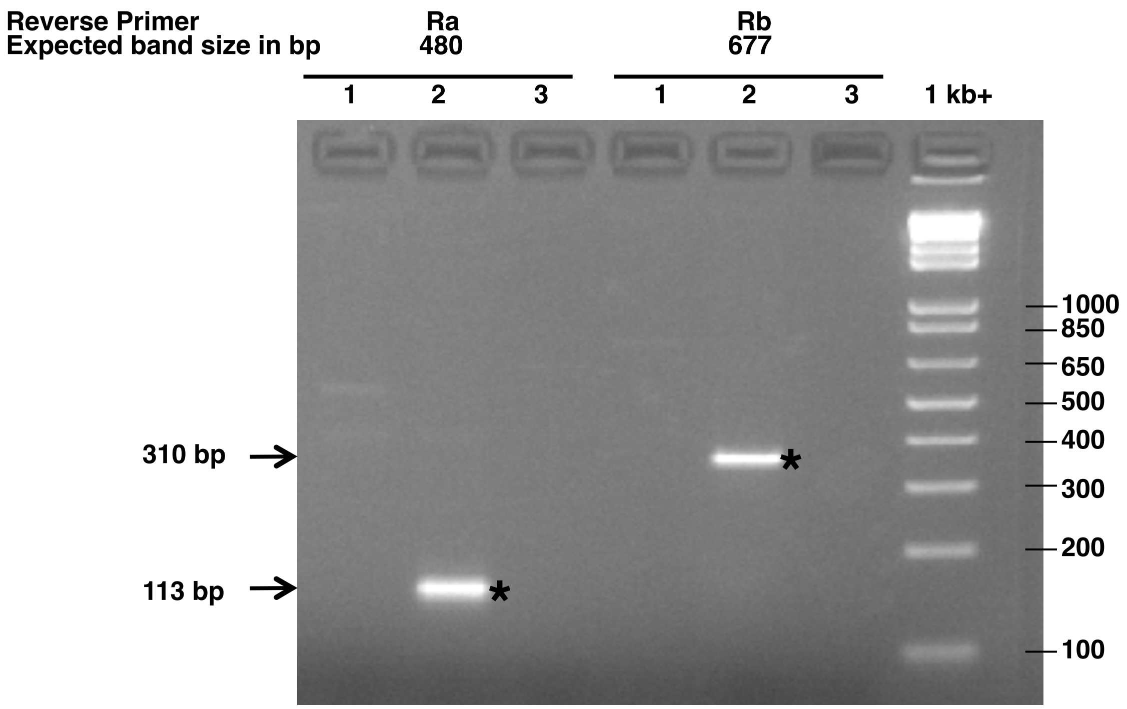

Additional File 3, Figure D

chrX:153260901-153261700

aacg**a**ttcggctgtgacgcgagcgcggccgctcccgatgcgctcgtgccg

cccccgccgtgctcctcggcagccgttg**ctCGGCCGGTTTTGGTAG**GCCC   **F**

GGGCCGCCGCCAGGCCTCCGCCTGAGCCCGCACCCGCCATGGACAACTAC   **Exon 1**

GC

(Breakpoint @ chrX:153,261,053 in exon 1)

AGATCTTTCGGATACCGAGCTGACCACCTTGCTGCGCCGGTACAACAT

CCCGCACGGGCCTGTAGTAGgtacgcggcggcgggcgggaccccttccgg

gccccctcctcgtgctccgcctcgcgacctccccgctgccctccccgcgc

gccttccccggcccgcggccctgaccgccccgtgtccggccag**GATCAAC**   **Exon 2**

TCGTAGGCTTTACGAGAAGAAGATCTTCGAGTACGAGACCCAGAGGCGGC

GGCTCTCGCCCCCCAGCTCGTCCGCCGCCTCCTCTTATAGCTTCTCTGgt

gagagcctcgcctgtggggacagcctgggacgcggggaggatggggtcgc

Gagggtgtggcagggggggc

(Breakpoint @ chrX:153,261,419 in intron 2)

cggtcgagagcggcactgg**agaaaggggagg**   **Ra**

gaagtctggggggcaaacagttctgtctcctcctttcaatccag**ACTTG**   **Exon 3**

AATTCGACTAGAGGGGATGCAGATATGTATGATCTTCCCAAGAAAGAGGA

CGCTTTACTCTACCAGAGCAAGGgtaaggcaggggttgggtgggcacgct   **Rb**

ggcaccttcacccgacttcgtcagggaccccgctc**acagggaggacctga**

gacctcagtcccaaccactccagcagccttaggagggagaaactgttaca
