# Supplementary material for: Detection limit of intragenic deletions with targeted array comparative genomic hybridization
Source: BMC Genet. 2013 Dec 5;14:116. doi: 10.1186/1471-2156-14-116 (PMC4235222; doi:10.1186/1471-2156-14-116)
Supplement: Additional file 4 — 12-bp deletion in the DBT gene. [file 1471-2156-14-116-S4.pdf]

Additional File 4

Figure A
Fragment analysis of breakpoint PCR of the 12-bp deletion within intron 5 of the *DBT* gene.
Figure shows an agarose gel with amplification of 12-bp deleted allele indistinguishable in size from wild type. Size ladder (Invitrogen 1 KB plus) is in the lane labeled as 1 kb+, while wild-type DNA in lane 1, proband DNA in lane 2, and water in lane 3. The two alternative reverse primers used in amplification are labeled as Ra and Rb. The expected sizes of fragments generated from normal wild type alleles are written on top of the gel. Asterisk highlights the band sequenced.

Figure B
Sequence from the deletion locus of the 12-bp deletion within intron 5 of the *DBT* gene.
Sequence shows the breakpoints in relation to the adjoining sequence; repeat elements, SNPs, exons, and primers. Sequence coordinates are listed according to UCSC hg 18 build (March 2006), and the two interruptions demarcate the breakpoints. Nucleotides in bold and capitalized font represent exons. Nucleotides that are underlined correspond to the primers used in breakpoint PCR. RepeatMasker are highlighted with blue font, SNPs (130 build) are highlighted in red font, and microhomology at breakpoints is shown boxed.

Additional File 4, Figure A

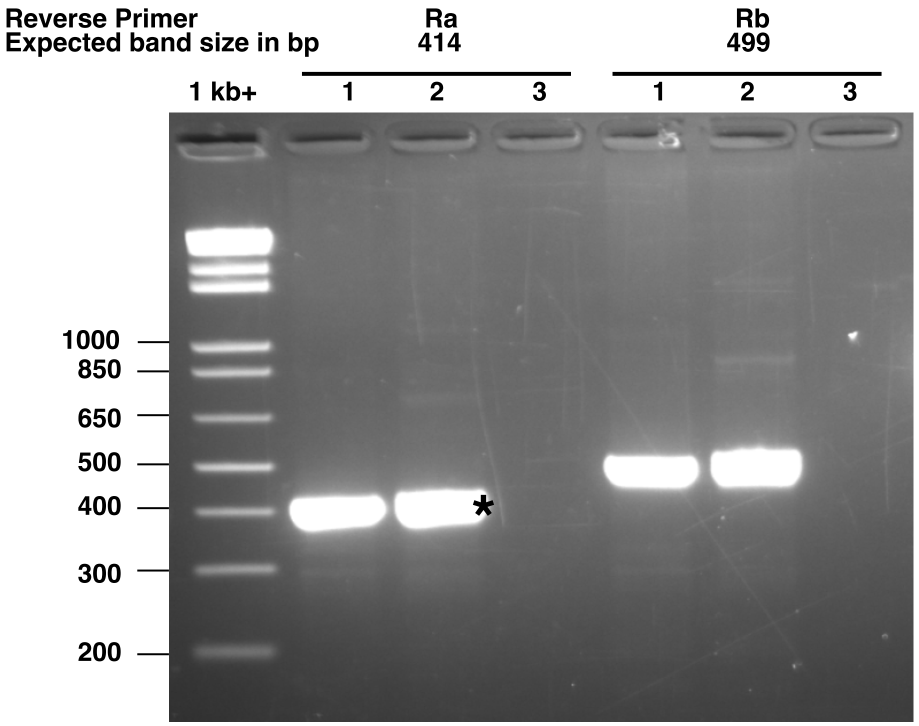

Additional File 4, Figure B

Chr1:100,456,701-100,457,150

gctaattttt**cagagatacaaatgtacacttccta**tacaatctcagactt **F**

aaatattaagagaacttac**ATTGTTTTCCATTGCCAGACGGCGAACTGCA** **Exon 5**

GGAGTTGCCAGTGTTTTTCGGCCCTTTATCTCTTGGTGTGTATGTTCATC

ATGAGACACTGCAGGAGTTTCAACAACATCTTCTTCTGAATctg

(Breakpoint @ chr1:100,456,895 in intron 4)

gtaaca

aggtaa

(Breakpoint @ chr1:100,456,906 in intron 4)

aacttaacttcagttgaaaaaaaattttttttttttactaagat

gtaaagtaaggccactaagatgtggcctataatatctaaataaattaaag

tttatagtctaaatgtataaaagttg**aactacttcagaataggaaaacac** **Ra**

aactatcctaatggtatgggtagagtggggggggggtgtgtgccggtac **Rb**

ctggtactt**tttaattattagtacctggtggatatgtca**ctcctgttgaa
