# Supplementary material for: Detection limit of intragenic deletions with targeted array comparative genomic hybridization
Source: BMC Genet. 2013 Dec 5;14:116. doi: 10.1186/1471-2156-14-116 (PMC4235222; doi:10.1186/1471-2156-14-116)

Additional File 5

Figure A
Fragment analysis of the Alu insertion in the *POMT1* gene.
Figure shows an agarose gel with wild type allele preferentially amplified over the allele with Alu insertion from the proband DNA. The white arrow points at the plausible larger insertion allele. An Alu specific primer F-Alu resulted in a patient-specific amplicon that was sequenced. Size ladder (Invitrogen 1 KB plus) is in the lane labeled as 1 kb+, while wild-type DNA in lane 1, proband DNA in lane 2, and water in lane 3. The alternative primer sets used in amplification are labeled on top. The expected sizes of fragments generated from normal wild type alleles are also written on top of the gel and the size of the amplicon generated from allele with insertion are highlighted with an arrow on the right. Asterisks highlight the bands sequenced.

Figure B
Electropherogram of exon 9/intron 9 boundary of *SLC9A6* showing hemizygous SNP that caused false deletion call on aCGH.
Sequence trace shows 3' end of exon 9 and adjoining intron 9 of the *SLC9A6* gene. Location of SNP is marked with black arrows. UCSC browser track showing base position.

Figure C
Electropherogram of compound heterozygous missense changes in the *GALT* gene that caused poor probe hybridization on aCGH.
Sequence trace shows exon 9 of the *GALT* gene. Location of the paternally inherited nucleotide change of unknown significance, and maternally is marked with black arrows. UCSC browser track showing base positions.

Additional File 5, Figure A

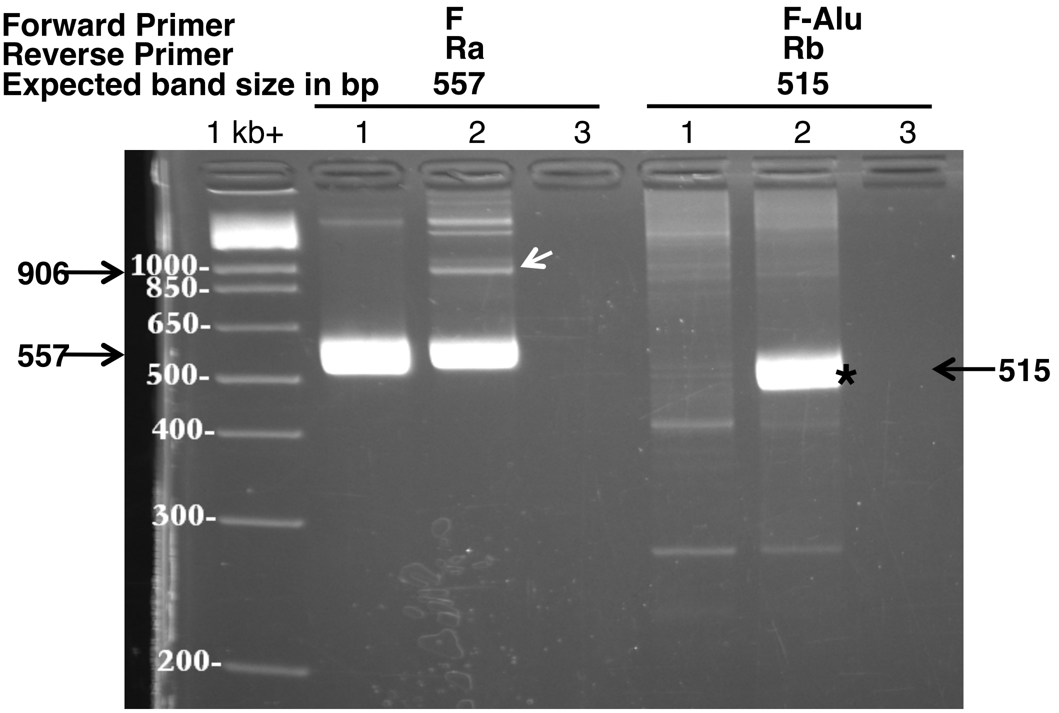

Additional File 5, Figure B

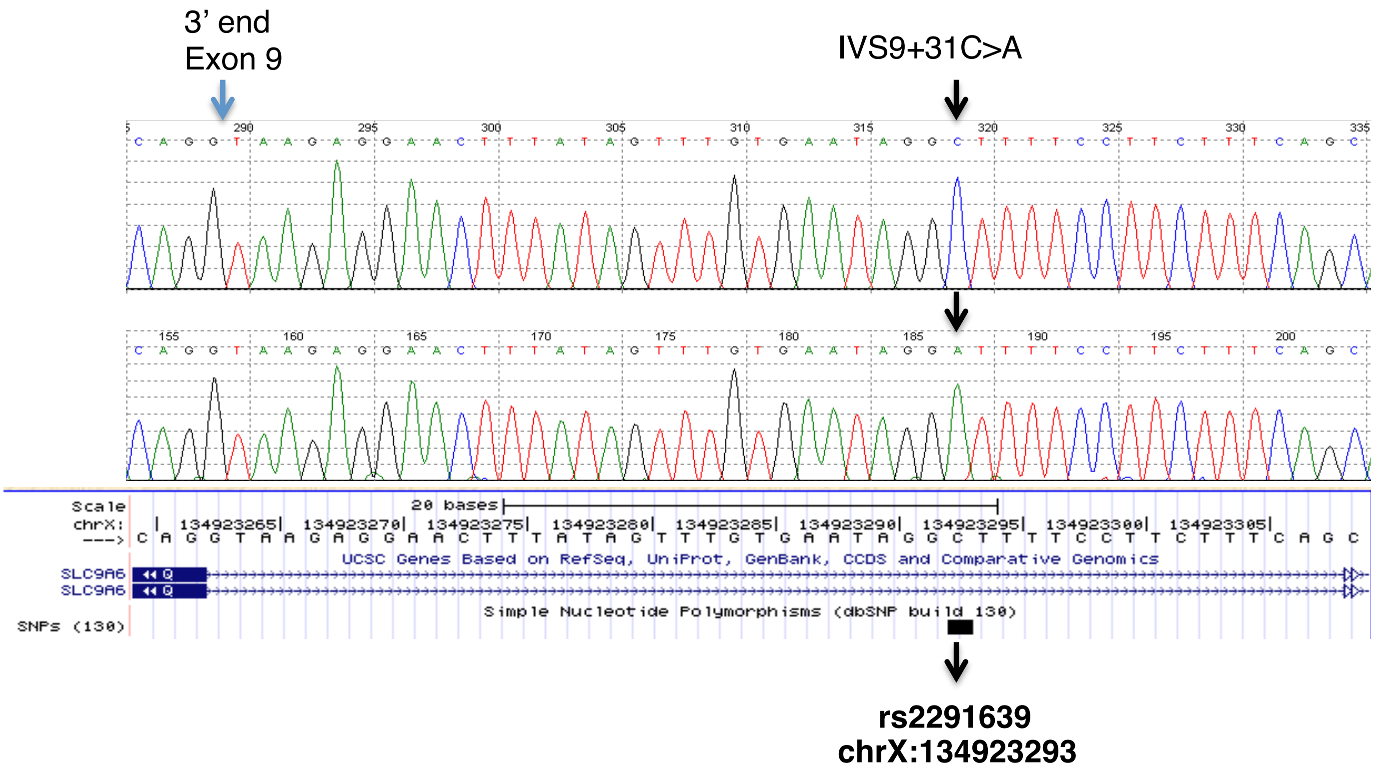

Additional File 5, Figure C

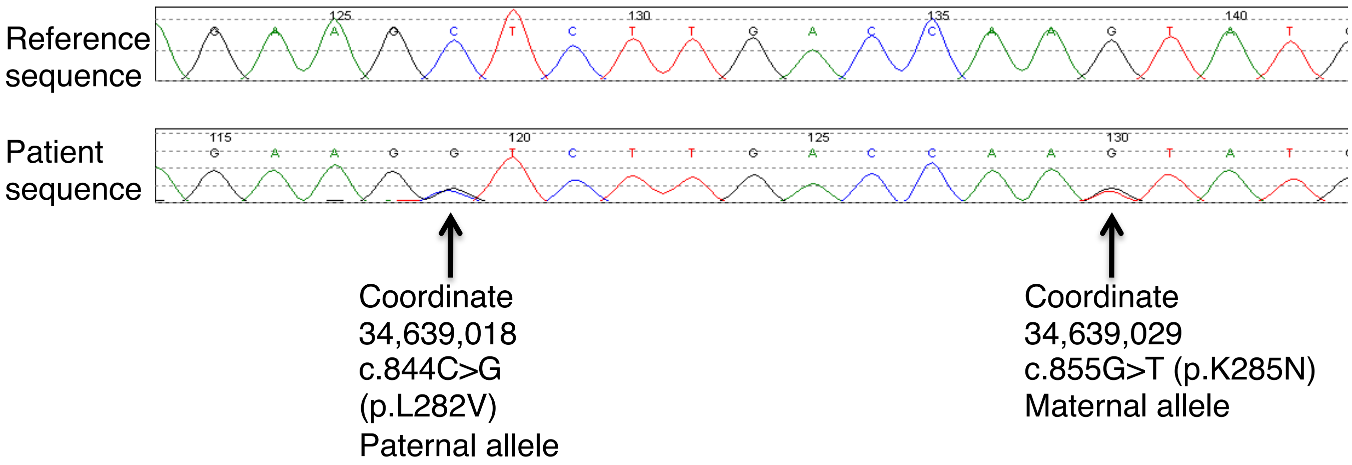

Supplement: Additional file 5 — False deletion calls in the POMT1 , SLC9A6 and GALT genes. [file 1471-2156-14-116-S5.pdf]
