# Supplementary material for: Detection limit of intragenic deletions with targeted array comparative genomic hybridization
Source: BMC Genet. 2013 Dec 5;14:116. doi: 10.1186/1471-2156-14-116 (PMC4235222; doi:10.1186/1471-2156-14-116)
Supplement: Additional file 6 — List of genes included in the custom-designed gene-targeted array. [file 1471-2156-14-116-S6.pdf]

Additional File 6

List of genes.

All array data discussed in this manuscript were generated using the custom-designed EGL_NMD_NBSplus_v1 array. Genes targeted in this array are listed below.

	EGL_NMD_NBSplus_v1 Gene Name
1	*ABCD1*
2	*ACSL4*
3	*ACTC1*
4	*AFF2*
5	*AGTR2*
6	*ALG1*
7	*ALG12*
8	*ALG2*
9	*ALG3*
10	*ALG6*
11	*ALG8*
12	*ALG9*
13	*ANK2*
14	*ANKS6*
15	*AP1S2*
16	*APC*
17	*ARHGEF6*
18	*ARHGEF9*
19	*ARX*
20	*ATP6AP2*
21	*ATP6V0A2*
22	*ATP7A*
23	*ATRX*
24	*B4GALT1*
25	*BBS1*
26	*BBS10*
27	*BBS12*
28	*BBS13*
29	*BBS14*
30	*BBS2*
31	*BBS3*
32	*BBS4*
33	*BBS5*
34	*BBS6*
35	*BBS7*
36	*BBS8*

**37**	***BBS9***
**38**	***BCOR***
**39**	***BICC1***
**40**	***BLM***
**41**	***BMPR1A***
**42**	***BRAF***
**43**	***BRCA1***
**44**	***BRCA2***
**45**	***BRWD3***
**46**	***CACNA1C***
**47**	***CACNA1S***
**48**	***CACNB2***
**49**	***CASK***
**50**	***CASQ2***
**51**	***CDKL5***
**52**	***CDKN1C***
**53**	***CDKN2A***
**54**	***CFTR***
**55**	***CHD7***
**56**	***COG1***
**57**	***COG7***
**58**	***COG8***
**59**	***COL4A3***
**60**	***COL4A4***
**61**	***COL4A5***
**62**	***CUL4B***
**63**	***CYP21A2***
**64**	***CYS1***
**65**	***DCX***
**66**	***DKC1***
**67**	***DLG3***
**68**	***DMD***
**69**	***DOLK***
**70**	***DPAGT1***
**71**	***DPM1***
**72**	***DPM3***
**73**	***DSC2***
**74**	***DSG2***
**75**	***DSP***
**76**	***EHMT1***
**77**	***ELK1***
**78**	***EPCAM / TACSTD1***
**79**	***FANCB***
**80**	***FGD1***

81	*FH*
82	*FLCN*
83	*FLNA*
84	*FMR1*
85	*FOXE1*
86	*FOXG1*
87	*FTSJ1*
88	*GCS1*
89	*GDI1*
90	*GJB2*
91	*GJB6*
92	*GK*
93	*GNE*
94	*GPC3*
95	*GPD1L*
96	*GRIA3*
97	*HBA1*
98	*HBA2*
99	*HCCS*
100	*HRAS*
101	*HSD17B10*
102	*HUWE1*
103	*IDS*
104	*IFT80*
105	*IGBP1*
106	*IKBKG*
107	*IL1RAPL1*
108	*INVS*
109	*IQSEC2*
110	*JARID1C*
111	*JUP*
112	*KCNE1*
113	*KCNE2*
114	*KCNH2*
115	*KCNJ2*
116	*KCNQ1*
117	*KIAA2022*
118	*KLF8*
119	*KRAS*
120	*L1CAM*
121	*LAMP2*
122	*LMBR1*
123	*MAGT1*
124	*MAOA*

**125**	***MAP2K1***
**126**	***MAP2K2***
**127**	***MBTPS2***
**128**	***MCKD1***
**129**	***MECP2***
**130**	***MED12***
**131**	***MEN1***
**132**	***MET***
**133**	***MGAT2***
**134**	***MID1***
**135**	***MLH1***
**136**	***MPDU1***
**137**	***MPI***
**138**	***MSH2***
**139**	***MSH6***
**140**	***MSK1***
**141**	***MTND1***
**142**	***MTND5***
**143**	***MTND6***
**144**	***MTTG***
**145**	***MTTH***
**146**	***MTTI***
**147**	***MTTK***
**148**	***MTTL1***
**149**	***MTTQ***
**150**	***MTTS1***
**151**	***MTTS2***
**152**	***MUTYH***
**153**	***MYBPC3***
**154**	***MYH7***
**155**	***MYL2***
**156**	***MYL3***
**157**	***NDP***
**158**	***NDUFA1***
**159**	***NEK8***
**160**	***NHS***
**161**	***NKX2-5***
**162**	***NLGN3***
**163**	***NLGN4X***
**164**	***NPHP1***
**165**	***NPHP3 / ACAD11***
**166**	***NPHP4***
**167**	***NPHP5***
**168**	***NRXN1***

**169**	***NSD1***
**170**	***NSDHL***
**171**	***NXF5***
**172**	***OCRL***
**173**	***OFD1***
**174**	***OPA1***
**175**	***OPA3***
**176**	***OPHN1***
**177**	***OTC***
**178**	***p53***
**179**	***PAK3***
**180**	***PAX8***
**181**	***PCDH19***
**182**	***PHF6***
**183**	***PHF8***
**184**	***PKD1***
**185**	***PKD2***
**186**	***PKHD1***
**187**	***PKP2***
**188**	***PLN***
**189**	***PLP1***
**190**	***PMS2***
**191**	***PORCN***
**192**	***PQBP1***
**193**	***PRKAG2***
**194**	***PRKCSH***
**195**	***PRPS1***
**196**	***PRSS1***
**197**	***PTEN***
**198**	***PTPN11***
**199**	***RAF1***
**200**	***RASA1***
**201**	***RECQL4***
**202**	***RET***
**203**	***RFT1***
**204**	***RPGRIP1L***
**205**	***RPL10***
**206**	***RPS6KA3***
**207**	***SCN1B***
**208**	***SCN4B***
**209**	***SCN5A***
**210**	***SEC63***
**211**	***SEMA4A***
**212**	***SHROOM4***

213	*SLC16A2*
214	*SLC35A1*
215	*SLC35C1*
216	*SLC6A8*
217	*SLC9A6*
218	*SMAD4*
219	*SMC1A / SMC1L1*
220	*SMS*
221	*SOS1*
222	*SOX3*
223	*SRPX2*
224	*STK11/LKB1*
225	*SUMF1*
226	*SYN1*
227	*SYP*
228	*TAZ*
229	*TIMM8A*
230	*TM4SF20*
231	*TMEM43*
232	*TMEM67*
233	*TNNC1*
234	*TNNI3*
235	*TNNT2*
236	*TPM1*
237	*TREM2*
238	*TRPC6*
239	*TSC1*
240	*TSC2*
241	*TSHR*
242	*TSPAN7*
243	*TTR*
244	*TUSC3*
245	*UBA5*
246	*UBE2A*
247	*UBE3A*
248	*UMOD*
249	*UPF3B*
250	*VCX3A*
251	*VHL*
252	*VWF*
253	*ZASP*
254	*ZCCHC12/SIZN2*
255	*ZDHHC15*
256	*ZDHHC9*

**257**	***ZEB2***
**258**	***ZNF41***
**259**	***ZNF674***
**260**	***ZNF711***
**261**	***ZNF81***
